# A Novel Modified Ultrasound-Guided Venipuncture Technique for Non-Tunneled PICC Insertion in a Non-Operating Room Anesthesia (NORA) Setting: A Technical Report with Real-World Experience

**DOI:** 10.3390/jcm15031234

**Published:** 2026-02-04

**Authors:** Dario Cirillo, Giorgio Ranieri, Gaetano Castellano, Domenico Pietro Santonastaso, Maria Silvia Barone, Isabella Russo, Antonio Coviello

**Affiliations:** 1 Department of Neurosciences, Reproductive and Odontostomatological Sciences, University of Naples “Federico II”, 80131 Naples, Italy; baronemariasilvia@gmail.com (M.S.B.); antonio_coviello@live.it (A.C.); 2 Complex Operational Unit of Anesthesia and Operating Units, Department of Emergency and Internal Medicine, Isola Tiberina Hospital, Gemelli Isola, 00168 Rome, Italy; gioranieri84@gmail.com; 3 Department of Anesthesia, Intensive Care and Pain Medicine, Sant’Ottone Frangipane Hospital, 83031 Ariano Irpino, Italy; castellanogaetano@me.com; 4 Anesthesia and Intensive Care Unit, AUSL Romagna, M. Bufalini Hospital, 47521 Cesena, Italy; domenicopietro.santonastaso@auslromagna.it; 5 Unit of Orthopedics and Traumatology, Department of Public Health, School of Medicine, University of Naples “Federico II”, 80131 Naples, Italy; isabella.russo@live.it

**Keywords:** peripherally inserted central catheter, PICC insertion, ultrasound-guided venipuncture, ultrasound-guided vascular access, non-tunneled central venous catheter, Seldinger technique, non-operating room anesthesia, NORA, vascular access complications, anesthesiology

## Abstract

**Background:** Peripherally inserted central catheters (PICCs) are widely used for medium- and long-term intravenous therapies but remain associated with mechanical and thrombotic complications, particularly during venipuncture and guidewire insertion. The growing use of Non-Operating Room Anesthesia (NORA) environments, where anesthesiologists frequently perform ultrasound-guided vascular access under conditions of limited resources and support, underscores the need for simple, reproducible, and inherently safe techniques. The objective of this technical note is to describe a modified ultrasound-guided venipuncture technique for non-tunneled PICC insertion, specifically developed for NORA settings, aimed at reducing procedure-related complications and preserving patient safety in routine clinical practice. **Methods:** The proposed technique consists of controlled intraluminal advancement of the needle tip (approximately 0.3–0.5 cm) under continuous ultrasound visualization, combined with progressive reduction in the insertion angle to achieve stable central intraluminal alignment before guidewire insertion. The technique has been applied in routine clinical practice across multiple Italian centers over the last two years, within a large multicenter real-world experience exceeding 5000 non-tunneled PICC procedures. **Results:** Based on real-world clinical observations, the systematic application of the technique was associated with a low incidence of early mechanical complications, including failed guidewire advancement, multiple venipuncture attempts, local pain, and hematoma formation. During standardized post-procedural ultrasound follow-up of the catheterized upper-extremity veins, no cases of catheter-related deep vein thrombosis were detected. **Conclusions:** This modified ultrasound-guided venipuncture technique represents a feasible and reproducible procedural refinement for non-tunneled PICC insertion in NORA environments. By enhancing intraluminal needle stability during guidewire advancement, it may contribute to improving procedural reliability and supporting patient safety in routine clinical practice. Further prospective and comparative studies are warranted to confirm these findings and define the generalizability of this approach.

## 1. Introduction

Peripherally Inserted Central Catheters (PICCs) are widely used for medium- and long-term intravenous therapies, including antimicrobial treatment, chemotherapy, and parenteral nutrition [1,2]. Their increasing utilization across hospital wards and outpatient settings has been accompanied by growing attention to procedure-related complications, which may occur immediately, early, or late after insertion [3].

National and International Consensus and Guidelines emphasize that the prevention of PICC-related complications begins before venipuncture [4,5,6,7]. The Rapid Peripheral Vein Assessment (RaPeVA) strategy promotes a systematic ultrasound evaluation of upper-arm veins, allowing for appropriate vein selection based on diameter, depth, compressibility, and relationship to surrounding anatomical structures [4,5,6,7,8]. Similarly, Dawson’s Zone Insertion Method provides a standardized anatomical framework to identify the optimal insertion site, minimizing mechanical stress and reducing the risk of thrombosis and catheter malfunction [9]. Current recommendations from the GAVeCeLT and other expert consensus documents strongly advocate for ultrasound-guided venous access, appropriate vein-to-catheter ratio, and meticulous adherence to standardized insertion protocols [10].

Despite these preventive strategies, PICC insertion remains associated with a non-negligible incidence of complications [11]. Several studies report rates of early mechanical complications—such as multiple venipuncture attempts, local pain, hematoma formation, and failed guidewire advancement—ranging from 10% to 30%, depending on patient characteristics and operator experience [11]. Catheter-related thrombosis, one of the most clinically relevant late complications, has been reported in up to 5–10% of cases, even when guideline-based preventive measures are applied [12,13,14].

Several phases of PICC insertion have been implicated in the development of complications [11,12,13,14]. While catheter dwell time and maintenance play a major role in late adverse events, the venipuncture and guidewire insertion phases appear to be particularly critical for immediate and early complications [4,5,6,7]. Inadequate needle tip positioning within the vessel lumen may lead to repeated puncture attempts, endothelial injury, vessel wall trauma, and resistance during guidewire advancement [11,12]. These factors can contribute to local complications such as pain and hematoma and may predispose patients to thrombotic events [13,14].

The conventional ultrasound-guided out-of-plane venipuncture technique, although widely adopted, may limit continuous visualization of the needle tip and result in marginal intraluminal positioning [4,13,14,15]. This technical limitation may be especially relevant in small-caliber veins or in patients with reduced venous compliance [8].

In recent years, the increasing demand for vascular access procedures outside the operating room has led to the widespread implementation of Non-Operating Room Anesthesia (NORA) environments [16]. In these settings, anesthesiologists are frequently involved in providing procedural sedation, monitoring, and ultrasound-guided vascular access for patients undergoing diagnostic or therapeutic interventions. However, unlike the operating room, NORA locations are often characterized by a relative lack of advanced monitoring systems, limited availability of specialized equipment, and reduced immediate access to support resources, which may increase the importance of technically simple, reproducible, and inherently safe procedural strategies [17]. Within this context, a simple, reproducible, and intrinsically safe ultrasound-guided technique for PICC placement may represent a valuable strategy to enhance procedural reliability and patient safety in daily clinical practice within the NORA setting.

Accordingly, the aim of this technical note is to describe a novel modified ultrasound-guided venipuncture technique for non-tunneled PICC insertion in NORA, designed to improve intraluminal needle stability during the critical phase of guidewire introduction and to potentially minimize complications associated with the conventional approach.

## 2. Materials and Methods

Over the last two years, this technique has been adopted in routine clinical practice across multiple Italian centers, including the University of Naples ‘Federico II’ (Naples, Italy), Isola Tiberina Hospital—Gemelli Isola (Rome, Italy), Sant’Ottone Frangipane Hospital (Ariano Irpino, Italy), and the Anesthesia and Intensive Care Unit of AUSL Romagna, M. Bufalini Hospital (Cesena, Italy).

In all centers, the technique has been routinely applied in the NORA environment by anesthesiologists–intensivists who are members of their institutional PICC teams, within a large multicenter real-world experience exceeding 5000 procedures.

No experimental intervention, randomization, or deviation from standard clinical care was introduced, and no identifiable patient data were collected or analyzed for research purposes.

### 2.1. Technique Description

All non-tunneled PICC insertions are performed in accordance with current GAVeCeLT recommendations and in full adherence to the SIP-2 protocol [4,15]. The patient is positioned supine with the arm abducted, and a systematic ultrasound assessment of the upper-arm veins is performed before venipuncture following the RaPeVA strategy. Vein diameter, depth, compressibility, and anatomical relationships are evaluated to identify the most suitable vessel and to anticipate potential technical difficulties. Vein selection follows guideline-based recommendations, with preference given to the basilic vein, followed by the brachial veins and, when appropriate, the cephalic vein. In obese patients or in cases of unfavorable basilic vein anatomy, the cephalic vein may be selected as the first-choice vessel due to its more superficial course and improved ultrasound accessibility. The venous diameter is measured in short-axis view without a tourniquet, and catheter size is selected to maintain an appropriate vein-to-catheter ratio, with the venous diameter being at least three times the external diameter of the catheter. The optimal insertion site is identified according to Dawson’s Zone Insertion Method, preferably within the proximal third of the arm. Before venipuncture, the brachial artery and median nerve are systematically identified using ultrasound to avoid inadvertent injury.

**Figure 1:**
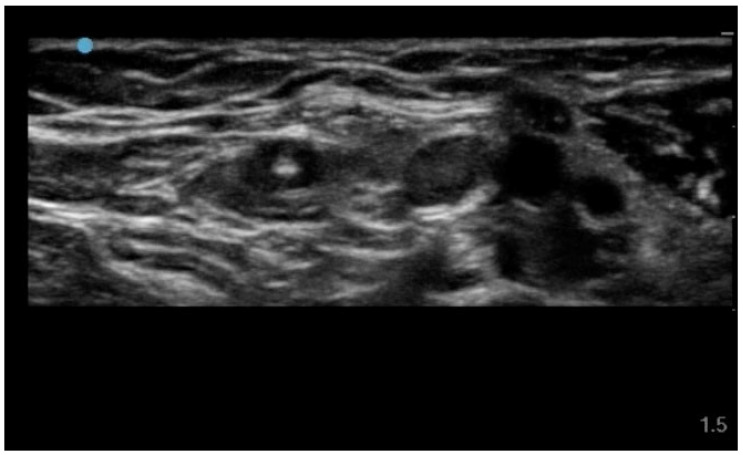
Ultrasound visualization of central intraluminal needle tip position.

Strict aseptic technique and maximal barrier precautions are applied in all cases. After skin antisepsis with 2% alcoholic chlorhexidine, local anesthesia is achieved by subcutaneous infiltration of 2–3 mL of 2% lidocaine. Venipuncture is then performed under real-time ultrasound guidance using an out-of-plane approach. Once the needle tip enters the vessel lumen, continuous visualization of the tip is maintained to ensure a central intraluminal position (Figure 1).

Real-time ultrasound image showing the needle tip clearly visualized and positioned at the center of the venous lumen. This central intraluminal alignment represents a key feature of the proposed technique, as it enhances needle stability and facilitates smooth advancement of the metallic Seldinger guidewire while reducing the risk of contact with the posterior vessel wall.

Unlike the conventional technique, the needle is gently advanced further into the vessel lumen by approximately 0.3–0.5 cm while maintaining constant ultrasound visualization through synchronous micro-adjustments of both the needle and the ultrasound probe. This controlled advancement aims to improve intraluminal needle stability during the most critical phase of the procedure.

**Figure 2:**
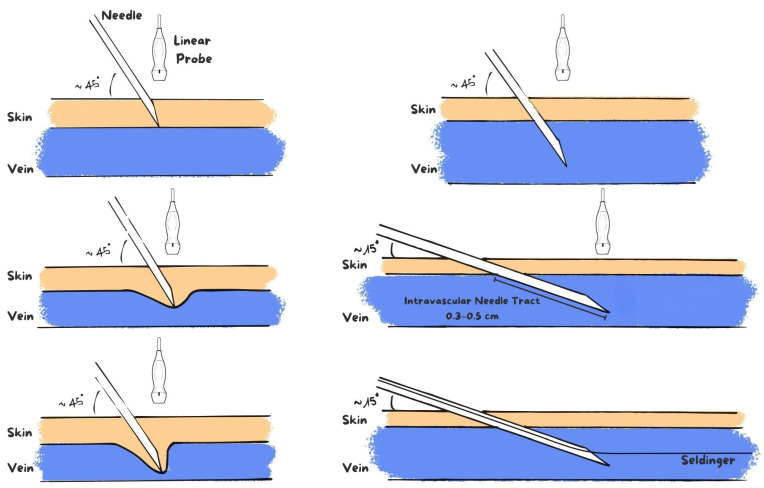
Novel modified ultrasound-guided venipuncture technique for PICC placement.

At this stage, particular attention is paid to maintaining the same needle inclination while the ultrasound probe is repositioned to allow guidewire insertion. Avoiding any change in the angle of the needle is essential to preserve the central alignment of the needle tip within the vessel lumen, thereby facilitating smooth advancement of the metallic Seldinger guidewire and reducing the risk of contact with the posterior vessel wall. Once stable intraluminal positioning is confirmed, the guidewire is introduced smoothly and without resistance (Figure 2).

Schematic illustration of the modified ultrasound-guided venipuncture technique described in this study. After entry of the needle tip into the venous lumen, the needle is gently advanced further intravascularly by approximately 0.3–0.5 cm, with a progressive reduction of the insertion angle (approximately 15°), while maintaining continuous ultrasound visualization through synchronous micro-adjustments of both the needle and the ultrasound probe. This controlled advancement improves intraluminal needle stability and promotes a more central alignment during Seldinger guidewire insertion.

**Figure 3:**
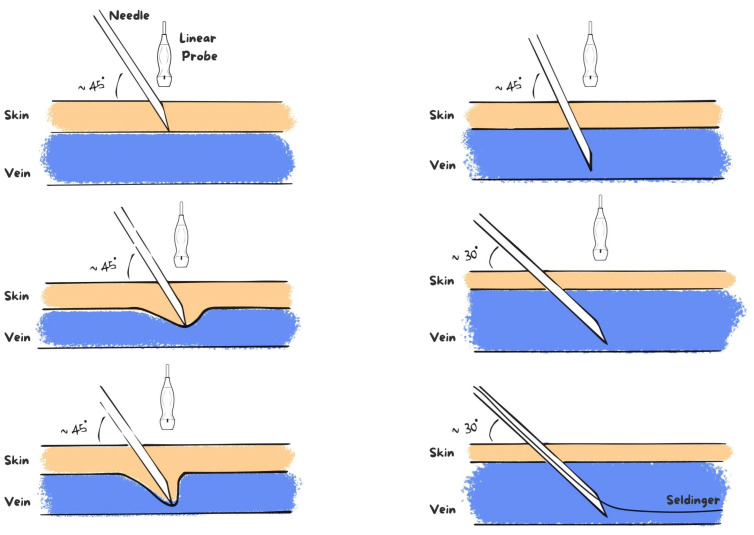
Conventional ultrasound-guided out-of-plane venipuncture technique.

A schematic summary of the main steps of the conventional venipuncture technique is presented in Figure 3 to allow appropriate comparison.

Schematic representation of the conventional ultrasound-guided out-of-plane venipuncture technique. The needle is introduced with an initial angle of approximately 45° until puncture of the anterior vessel wall; the needle tip enters the venous lumen with limited intraluminal advancement and often remains marginal rather than centrally aligned. This configuration may reduce intraluminal needle stability during guidewire insertion and increase the risk of contact with the posterior vessel wall.

After additional local anesthetic infiltration at the catheter exit site, a small skin incision is performed, followed by insertion of the microintroducer–dilator system according to standard technique.

Subsequent catheter advancement and tip navigation are performed under ultrasound guidance of the supraclavicular region, with routine visualization of the ipsilateral internal jugular vein to facilitate correct catheter progression and to promptly identify malposition. Final catheter tip location is confirmed using intracavitary ECG guidance. After correct positioning is achieved, the PICC is secured with a sutureless fixation device, the exit site is sealed with cyanoacrylate tissue adhesive, and a semipermeable transparent dressing is applied to complete the procedure.

### 2.2. Real-World Clinical Experience

Despite adherence to guideline-recommended preventive strategies, non-tunneled PICC insertion remains associated with a non-negligible incidence of early mechanical and thrombotic complications [11,13,14]. Published data report variable rates of periprocedural adverse events, including local hematoma or bleeding (6–27%), pain at the puncture site (10–20%), failed venipuncture or guidewire advancement (2–3%), and catheter-related thrombosis, reported in 6–14% of cases, with symptomatic deep vein thrombosis occurring in approximately 1.5–6.7% of patients [18,19,20]. Higher rates have been described in selected populations, particularly oncologic and critically ill patients, and asymptomatic thrombosis has been reported in up to 72% of cases in studies employing systematic ultrasound screening [3,4,21].

Within this context, the modified ultrasound-guided venipuncture technique described in this technical note has been systematically applied over the last two years in routine clinical practice across multiple Italian centers, within a large multicenter real-world experience exceeding 5000 non-tunneled PICC procedures. All procedures were directly performed by anesthesiologists–intensivists actively involved in their local PICC teams, with specific expertise in ultrasound-guided vascular access and in full adherence to institutional protocols and international recommendations. After its initial introduction, the technique was rapidly learned and consistently reproduced by other members of the PICC teams across the participating centers, without the need for additional equipment or substantial modification of standard workflows, supporting its feasibility and reproducibility in routine clinical settings.

**Figure 4:**
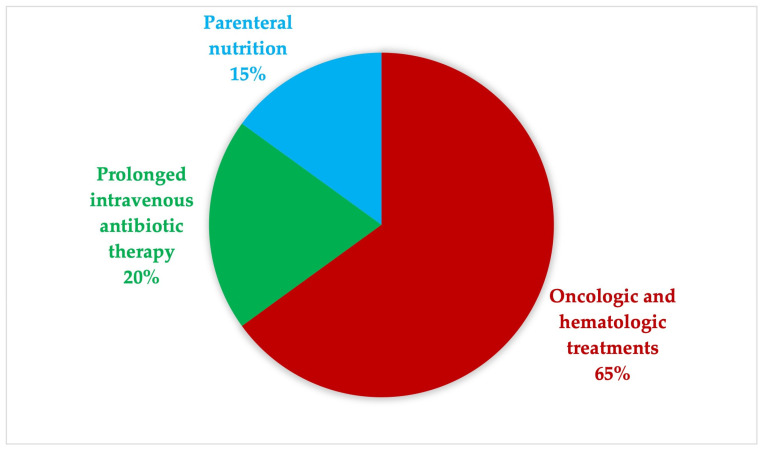
Distribution of the main indications for non-tunneled PICC placement.

As shown in Figure 4, PICCs were placed for a broad range of indications, predominantly in oncologic and hematologic patients, who accounted for 65% of all procedures. Additional indications included prolonged intravenous antibiotic therapy (20%) and parenteral nutrition (15%). Critically ill patients were excluded from the present analysis, in order to focus on the NORA environment and on elective or semi-elective procedural contexts.

Importantly, the introduction of this procedural refinement did not result in a clinically relevant prolongation of procedural time when compared with our institutional standards established during the period preceding its implementation, when the conventional technique was routinely applied. Based on internal benchmarking and operator feedback, the overall duration of PICC placement remained comparable to that observed in the earlier institutional experience.

Intraluminal needle stability was not assessed using predefined quantitative metrics but was evaluated qualitatively during real-time ultrasound guidance. Stability was inferred from the ability to maintain a central intraluminal needle tip position while repositioning the ultrasound probe and during guidewire insertion, without visible needle displacement, posterior vessel wall contact, or resistance to guidewire advancement.

Based on this cumulative clinical experience, the incidence of complications commonly reported in the literature appeared markedly lower. Although no formal first-attempt success rate was prospectively recorded, operators consistently reported a high rate of successful guidewire advancement at the initial venipuncture, with virtually no need for repeated puncture attempts. In line with these observations, patient-reported pain at the insertion site was minimal or transient in the vast majority of procedures. Local adverse events such as hematoma or clinically relevant bleeding were rare.

Given that catheter-related thrombosis may frequently be asymptomatic, all patients underwent systematic ultrasound examination of the catheterized upper-extremity veins during a standardized post-procedural follow-up, in accordance with accepted recommendations for venous thromboembolism [22]. Within this structured imaging-based surveillance, no catheter-related deep vein thrombosis was detected, corresponding to a proportion of 0 cases within the observed real-world clinical experience. This systematic imaging-based surveillance allowed accurate assessment of thrombotic events and exclusion of clinically silent thrombosis. The absence of detected thrombosis supports the hypothesis that reducing endothelial trauma during venipuncture and guidewire insertion may play a role in lowering thrombotic risk.

Overall, these real-world observations suggest a favorable safety profile of the modified technique with respect to both technical success and early procedure-related complications in a NORA environment. Although no formal statistical analysis was performed and no direct comparison with the conventional technique was undertaken, the consistently low incidence of adverse events provided the rationale for formally describing this procedural refinement in the present technical note.

## 3. Discussion

This technical note describes a modified ultrasound-guided venipuncture technique for non-tunneled PICC insertion specifically applied in a NORA setting, where anesthesiologists are increasingly involved in ultrasound-guided vascular access as part of perioperative and procedural care outside the operating room. Over the last two years, this technique has been systematically implemented across multiple centers, based on a large multicenter real-world experience exceeding 5000 procedures, predominantly in oncologic and hematologic patients requiring chemotherapy or prolonged intravenous therapies. Within this real-world NORA environment, the proposed modification was associated with a consistently low incidence of early mechanical complications, including failed guidewire advancement, multiple venipuncture attempts, local pain, and hematoma formation, compared with complication rates commonly reported in the literature. These findings suggest that targeted optimization of the venipuncture and guidewire insertion phases in ultrasound-guided non-tunneled PICC placement may represent a pragmatic technical refinement, potentially promoting reliable vascular access and patient safety in routine NORA practice.

NORA represents a rapidly expanding area of anesthetic practice, increasingly involving patients with significant comorbidities and procedures performed in environments that often lack the standardization, equipment availability, and immediate support typical of the operating room [16]. These system-related constraints have been consistently associated with a higher risk of preventable adverse events, underscoring the importance of workflow optimization and technically reliable approaches [16,17]. Recent narrative reviews and safety frameworks emphasize that environmental factors, unfamiliar equipment, limited monitoring, and organizational variability may predispose to procedural complications, while structured protocols and the use of ultrasound guidance are key elements for risk mitigation in NORA settings [23,24]. In parallel, contemporary guidelines on safe vascular access advocate routine ultrasound guidance and standardized insertion practices to improve success rates and reduce mechanical complications [7,25]. Within this framework, our technical refinement—aimed at enhancing intraluminal needle stability during the critical venipuncture and guidewire insertion phases—may be particularly relevant in NORA environments, where simplicity, reproducibility, and intrinsic procedural safety are essential to support reliable vascular access in daily clinical practice.

Compared with the conventional ultrasound-guided out-of-plane venipuncture technique, the proposed modification primarily addresses limitations related to needle angulation and intraluminal stability during guidewire insertion. In the conventional approach, venous access is typically achieved with a relatively steep needle inclination (approximately 45°), and needle visualization is limited to a hyperechoic dot representing the intersection of the needle with the ultrasound beam. Once blood return confirms intraluminal entry, needle advancement is usually halted, and the Seldinger guidewire is introduced from a marginal and often angled intravascular position. This configuration may predispose to posterior vessel wall contact, resistance during guidewire advancement, and endothelial trauma, particularly in small-caliber or low-compliance veins [4,13,14,15].

In contrast, the novel modified technique emphasizes precise pre-procedural vessel centering, continuous dynamic needle tip positioning, and controlled intraluminal needle advancement after venous entry. By gently advancing the needle further into the vessel lumen while maintaining ultrasound visualization and reducing the insertion angle, a more stable and central intraluminal alignment is achieved before guidewire insertion. This refinement facilitates smoother guidewire progression and may reduce mechanical stress on the vessel wall, providing a plausible mechanistic explanation for the lower incidence of early mechanical complications observed in real-world clinical practice.

A relevant point of clarification concerns the distinction between the proposed technique and in-plane (long-axis) ultrasound-guided cannulation. The in-plane approach enables continuous visualization of the needle shaft and tip along the longitudinal axis of the vessel and is recommended in current guidelines when precise depth control is required [8]. However, maintaining stable long-axis alignment of the vessel while advancing the needle entirely in-plane can be technically challenging, particularly in adult patients, deeper veins, or anatomically complex regions [8]. Conversely, the conventional out-of-plane (short-axis) approach is intentionally designed to maintain visualization of surrounding anatomical structures, such as the brachial artery or median nerve, during brachial vein cannulation, which may enhance procedural safety in anatomically crowded areas. Nevertheless, limited intraluminal needle stability and suboptimal angulation during guidewire insertion remain recognized limitations of the conventional short-axis technique.

The modified technique described in this technical note is not intended to replace the in-plane approach nor to establish superiority over existing methods. Rather, it represents a pragmatic refinement of the traditional out-of-plane technique, aiming to improve intraluminal needle alignment and stability during the critical guidewire insertion phase while preserving continuous visualization of adjacent structures. In accordance with the Recommendations of the American Society of Echocardiography, safe ultrasound-guided cannulation relies on direct needle tip visualization irrespective of the selected approach, and the choice between in-plane and out-of-plane techniques should remain guided by operator experience, vessel anatomy, and clinical context [8].

Within this conceptual framework, the degree of vascular traumaticity associated with catheter insertion appears to represent a key determinant of catheter performance, independently of the specific ultrasound approach adopted. This interpretation is further supported by recent evidence emphasizing the role of insertion-related trauma in catheter outcomes [26]. In a large pediatric intensive care cohort, Armstrong et al. [26] reported a limited mean dwell time of approximately 50 h for peripheral intravascular catheters and identified patient agitation, tissue edema, and irritant infusions as major predictors of early catheter failure. Notably, the authors highlighted how mechanical stress, repeated manipulation, and endothelial injury significantly contribute to reduced catheter longevity and increased complication rates [26].

Although conducted in a different clinical setting and involving peripheral catheters rather than PICCs, these findings reinforce the concept that minimizing vascular trauma at the time of insertion represents a critical determinant of catheter-related outcomes. Within this framework, the reduced traumaticity associated with the modified ultrasound-guided venipuncture technique described in the present study may have contributed to the consistently low incidence of early mechanical and thrombotic complications observed in routine clinical practice.

Several alternative approaches to ultrasound-guided PICC insertion have been described in the literature with the aim of improving procedural success and reducing complications, particularly those related to venipuncture and guidewire advancement.

In a recent prospective observational study, Li et al. [19] investigated refinements of ultrasound-guided venous access techniques aimed at improving needle visualization and first-attempt success during PICC placement. The authors emphasized the importance of continuous needle tip visualization and reported improved procedural metrics; however, their approach primarily focused on ultrasound handling and operator ergonomics rather than on deliberate intraluminal needle advancement. While effective in experienced hands, this strategy may still be limited by marginal needle positioning during guidewire insertion, particularly in small-caliber veins [19].

Similarly, Villa et al. [27] reported a modified ultrasound-guided vascular access strategy in a multicenter observational study involving critically ill patients. Their work highlighted the role of standardized ultrasound protocols and operator training in reducing mechanical complications. However, the technique did not specifically address intraluminal needle stabilization during the guidewire insertion phase, which remains a frequent source of resistance, endothelial trauma, and procedural failure [27].

Another relevant contribution comes from Takeshita et al. [28], who evaluated different ultrasound-guided venipuncture approaches in a comparative clinical study. Although improved visualization techniques were associated with higher first-pass success rates, the study did not explore controlled intravascular needle advancement as a strategy to enhance guidewire stability. Moreover, reported outcomes were largely limited to technical success rather than early thrombotic or bleeding complications [28].

In contrast, the technique described in the present technical note introduces a deliberate and controlled advancement of the needle tip further into the vessel lumen (approximately 0.3–0.5 cm) under continuous ultrasound visualization. This refinement is specifically designed to enhance intraluminal needle stability during guidewire insertion, potentially reducing posterior wall contact, endothelial injury, and the need for repeated puncture attempts. Importantly, this modification does not require additional equipment, changes in patient positioning, or deviation from established ultrasound-guided vascular access workflows.

An additional aspect of interest concerns the potential applicability of this technique in patients at increased risk of bleeding, such as those with thrombocytopenia or receiving anticoagulant therapy. These populations are known to be particularly vulnerable to local complications related to venipuncture, including hematoma formation and prolonged bleeding.

Available evidence suggests that ultrasound-guided venous access is associated with a lower incidence of bleeding complications in patients with coagulation abnormalities. Zhang et al. reported favorable safety outcomes for central venous access in thrombocytopenic patients when ultrasound guidance and atraumatic techniques were employed [29]. Similarly, earlier observational studies by Potet et al. demonstrated that ultrasound-guided venipuncture reduces vascular trauma and bleeding risk in anticoagulated patients compared with landmark-based techniques [30].

Furthermore, Scamuffa et al. [31] highlighted that minimizing endothelial injury during venous access may also play a role in reducing thrombotic risk, particularly in patients with altered hemostasis. Within this framework, the enhanced intraluminal stability provided by the modified technique described herein may theoretically offer additional safety benefits by limiting vessel wall trauma during guidewire advancement [31].

Although the present work does not include a dedicated analysis of thrombocytopenic or anticoagulated patients, the consistently low incidence of local bleeding, hematoma formation, and thrombotic events observed in real-world practice suggests that this approach may be particularly well suited for high-risk populations. Nevertheless, this hypothesis remains speculative and warrants confirmation through prospective studies specifically designed to evaluate safety outcomes in these patient subsets.

Several limitations must be acknowledged. The clinical experience reported in this technical note is retrospective and non-comparative, and no formal statistical analysis was performed. Procedural efficacy outcomes—including first-attempt success rate and guidewire advancement success—were not prospectively defined or collected as predefined endpoints, but rather derived from routine real-world clinical observations.

Intraluminal needle stability was assessed qualitatively under real-time ultrasound guidance rather than through objective quantitative metrics, representing an inherent limitation of the present technical note.

Potential confounding factors, including operator expertise, patient characteristics, and vein anatomy, were not systematically controlled. Moreover, despite the use of a standardized ultrasound-based follow-up, subclinical thrombotic events occurring outside the predefined follow-up window cannot be completely excluded.

As a technical note, this article does not aim to demonstrate superiority over existing techniques but rather to describe a procedural refinement that appears feasible, reproducible, and safe in routine clinical practice. Future prospective, multicenter comparative studies are needed to evaluate the reproducibility of this technique across operators with varying levels of experience and to assess its impact on both early and late PICC-related complications, particularly in patients at increased bleeding or thrombotic risk.

## 4. Conclusions

The modified ultrasound-guided venipuncture technique described in this technical note represents a feasible and reproducible refinement of the conventional approach for non-tunneled PICC insertion in the NORA setting. By enhancing intraluminal needle stability during venipuncture and guidewire advancement, this technique may contribute to improving procedural reliability and supporting patient safety, as suggested by multicenter real-world clinical experience. In the context of the expanding role of anesthesiologists in ultrasound-guided vascular access outside the operating room, this approach may offer a pragmatic option for daily NORA practice. However, given the retrospective and non-comparative nature of the present observations, further prospective and comparative studies are warranted to better define its clinical impact, reproducibility, and generalizability across different operators and patient populations.

## Data Availability

No new data were created or analyzed in this study. Data sharing is not applicable to this article.
